# Machine vision model using nail images for non-invasive detection of iron deficiency anemia in university students

**DOI:** 10.3389/fdata.2025.1557600

**Published:** 2025-04-09

**Authors:** Jorge Raul Navarro-Cabrera, Miguel Angel Valles-Coral, María Elena Farro-Roque, Nelly Reátegui-Lozano, Lolita Arévalo-Fasanando

**Affiliations:** ^1^Faculty of Systems Engineering and Computer Science, Universidad Nacional de San Martín, Tarapoto, Peru; ^2^Faculty of Health Sciences, Universidad Nacional de San Martín, Tarapoto, Peru; ^3^Faculty of Human Medicine, Universidad Nacional de San Martín, Tarapoto, Peru

**Keywords:** cross validation, data capture, DenseNet169, deep learning, non-invasive diagnostics

## Abstract

**Introduction:**

Iron deficiency anemia (IDA) is a global health issue that significantly affects quality of life. Non-invasive methods, such as image analysis using artificial vision, offer accessible alternatives for diagnosis. This study proposes a DenseNet169-based model to detect anemia from nail images and compares its performance with that of the Rad-67 hemoglobin meter.

**Methods:**

A cross-sectional study was conducted with 909 nail images collected from university students aged 18–25 years at the Universidad Nacional de San Martín, Peru. Samsung Galaxy A73 5G was used to capture images under controlled conditions, and clinical data were complemented with hemoglobin readings from the Rad-67 device. The images were pre-processed using segmentation and data augmentation techniques to standardize the dataset. Three models (DenseNet169, InceptionV3, and Xception) were trained and evaluated using metrics, such as accuracy, recall, and AUC.

**Results:**

DenseNet169169 demonstrated the best performance, achieving an accuracy of 0.6983, recall of 0.6477, F1-Score of 0.6525, and AUC of 0.7409. Despite the presence of false-negatives, the results showed a positive correlation with Rad-67 readings.

**Conclusion:**

The DenseNet169-based model proved to be a promising tool for non-invasive detection of iron deficiency anemia, with potential for application in clinical and educational settings. Future improvements in preprocessing and dataset diversification could enhance performance and applicability.

## 1 Introduction

According to the World Health Organization (WHO, 2023), anemia is one of the most prevalent blood disorders worldwide, significantly impacting public health. Clinically, anemia is defined as a condition in which the number of red blood cells or the concentration of hemoglobin (Hb) falls below normal levels (Saboor et al., 2021; Las Heras Manso, 2022). This deficiency results in symptoms such as fatigue, weakness, dizziness, and shortness of breath, which can severely affect an individual's quality of life (Camaschella, 2019). Anemia is commonly associated with nutritional deficiencies, chronic diseases, genetic disorders, and certain medications or treatments (Deivita et al., 2021).

The detection and proper diagnosis of anemia remain a major global challenge due to its socio-economic implications. The costs associated with anemia management, including prevention, diagnosis, and treatment, place a significant burden on healthcare systems (Amegbor et al., 2022). The most common diagnostic approach involves invasive blood tests, which can cause discomfort or fear in patients. Additionally, non-invasive detection methods remain financially inaccessible for developing countries due to their high market cost (An et al., 2021; Ramaswamy et al., 2021).

As a result, recent studies have explored artificial intelligence (AI) and computer vision as non-invasive alternatives for estimating hemoglobin levels. This research trend focuses on analyzing images of nail beds, fingertips, and palms to determine anemia status (Ghosal et al., 2022; Peksi et al., 2021; Selvi et al., 2022; Yilmaz et al., 2022; Das et al., 2023; Asare et al., 2023). These AI-driven approaches offer promising, cost-effective, and accessible solutions for anemia diagnosis. However, current models remain at the prototype level and require improved accuracy and reliability before widespread adoption (Dimauro et al., 2020, 2023; Saavedra Grandez, 2021).

Despite these advancements, a notable limitation in existing studies is their narrow focus on specific populations, particularly children and pregnant women. While these groups have a high prevalence of anemia and represent at-risk populations (Stevens et al., 2022), few studies have investigated anemia detection in other demographics, such as young adults and university students. This group is also susceptible to anemia, which can significantly impact cognitive function, academic performance, and daily activities (Hamali et al., 2020; Khani Jeihooni et al., 2021; Stevens et al., 2022).

In the Peruvian context, anemia has become a growing concern, particularly in the San Martín region, where the Ministerio de Desarrollo e Inclusión Social (2023) reported a 10% increase in anemia cases among young adults over the past 2 years. This issue particularly affects university students, placing them in a high-risk category. According to AlJaber et al. (2019) and Choi (2020), university students experience additional challenges such as academic stress, poor diet, and unhealthy lifestyle habits, which may contribute to iron deficiency anemia. The presence of anemia in this population can negatively affect concentration, energy levels, and overall academic performance (Amoaning et al., 2022).

Given these challenges, there is a need to develop novel, cost-effective, and non-invasive approaches that leverage machine learning and computer vision to detect anemia. Therefore, this study proposes a deep learning-based computer vision model for classifying images of university students' fingernails. This approach takes advantage of the availability of the Rad-67 hemoglobin meter at the National University of San Martín, Peru, using its readings as ground truth to evaluate model performance.

Unlike previous studies that primarily focus on hemoglobin estimation through conjunctiva or palm-based imaging, this research introduces a systematic evaluation of transfer learning architectures applied exclusively to fingernail images for anemia detection. The contribution of this study lies in its comparative analysis of three state-of-the-art deep learning models—InceptionV3, DenseNet169169, and Xception—assessing their classification performance and potential for clinical applications. Additionally, the dataset used is one of the first to explore fingernail-based anemia detection in a university population, providing new insights into the feasibility of non-invasive diagnostic approaches.

## 2 Related jobs

Over the last decade, the field of biomedical data analysis has experienced substantial advancements, laying the foundation for current studies. Consequently, this section reviews the non-invasive methods previously used for anemia detection. In Indonesia, Peksi et al. (2021) aimed to achieve early anemia detection by analyzing nail and palm images using the Naive Bayes method. The system was developed using the cascade method. The results indicated an anemia detection accuracy of 87.5% under varying light intensities, which increased to 92.3% at a light intensity of 5362 lx. The novelty of this study lies in the pre-processing and image classification methods employed. The nail and palm images were converted into the YCbCr color space for segmentation and color feature extraction. These features were then classified using the Naive Bayes method. The system classifies the input images as either normal or anemic.

In Turkey, Yilmaz et al. (2022) proposed a combined deep learning methodology to non-invasively estimate blood hemoglobin levels. For the estimation, they utilized data such as age, height, weight, body mass index, sex, and nail images. The deep-learning model combines a numerical data model with a nail image model. The study bias was calculated as 0.03 g/dL, with concordance limits at a 95% confidence interval calculated as 1.09 g/dL. The mean absolute percentage error was 2.09%, and the root mean square error was 0.56 g/dL. The average response time were 0.09 s. The results demonstrated the study's success compared to similar studies.

In India, Ghosal et al. (2022) introduced a model called iNAP based on the Internet of Medical Things (IoMT), to address the limitations in detecting anemia and polycythemia. The model uses a smartphone camera to capture images of the eyes (conjunctiva) and nails as the regions of interest. The algorithm analyzes the color spectroscopy of these regions, extracts the dominant color, and accurately predicts hemoglobin levels. Anemia is classified as a hemoglobin value below 11.5 g/dL, whereas polycythemia is classified as a value above 16.5 g/dL. The model predicts blood hemoglobin levels with an accuracy of ±0.33 g/dL, a bias of 0.2 g/dL, and a recall of 90% compared to clinically tested results in 99 participants.

In the same country, Selvi et al. (2022) highlighted the impact of micronutrient deficiencies on overall health by analyzing nail color and metadata using a proposed deep learning model. This model employs convolutional neural network (CNN) architectures, such as ResNet, SqueezeNet, DenseNet169, VGG, and a custom model, to extract key features and predict deficiencies. The model was trained with real-time images and validated using sample images to improve accuracy. The non-invasive model achieved an accuracy of 94%. This predictive mechanism can be useful in primary healthcare settings and is connected to frontline workers for quick and easy diagnosis.

In a related context, Das et al. (2023) aimed to combine state-of-the-art artificial intelligence techniques with conventional practices to assess anemia by observing the nail pallor. The proposed method induces color changes in the nail bed by applying and releasing pressure using a custom hardware device. Videos captured with a smartphone were analyzed to measure the color change rate and quantify the blood hemoglobin levels by correlating them with clinically determined values. The proposed prediction model, based on a fusion approach, outperformed existing solutions, demonstrating mean RMSE and MSE errors of 0.63 and 0.61, respectively, and standard deviations of 0.46 and 0.73 g/dL compared to clinically tested hemoglobin levels in a group of 220 individuals.

In Ghana, Asare et al. (2023) applied various machine learning algorithms, including Naive Bayes, CNN, SVM, k-NN, and decision trees, to detect iron deficiency anemia. They compared images of the eye conjunctiva, palpable palms, and nail color to determine the most accurate method for detecting anemia in children. This study consisted of three stages: dataset collection, dataset preprocessing, and model development for anemia detection. CNN achieved the highest accuracy at 99.12%, whereas SVM had the lowest accuracy at 95.4%. These results demonstrate the effectiveness of the non-invasive approach in anemia detection, supporting its potential as an efficient diagnostic mechanism.

Over the past decade, the field of computer vision and biomedical image analysis has experienced significant growth, laying the groundwork for current studies on non-invasive disease detection. Although various approaches have been developed to detect anemia using biomedical images, it is crucial to note that most studies have not focused on specific populations, such as university students, who may present with particular risk factors. In this context, using nail images as an indicator of iron deficiency anemia has emerged as an innovative and relevant alternative, especially for young populations in educational settings.

Most previous studies on anemia detection have utilized nail image analysis and implemented deep learning models, particularly CNNs, to develop non-invasive methods. However, there is a continued need to optimize the performance of these methodologies by incorporating new and accessible datasets and adopting standardized protocols for image capture. Additionally, there is a marked lack of research focused on young populations, such as university students, who are at a significant risk of iron deficiency anemia but have been underrepresented in prior studies. In this context, our study proposes the application of a DenseNet169169-based model, which is one of the first to use this advanced architecture for anemia detection in university students. This approach aims to address the existing research gap and offers a non-invasive, innovative, and effective solution tailored to this vulnerable demographic group.

## 3 Proposed method

In this section, we describe the stages and procedures implemented in our study, structuring the methodology into four main phases based on the research of Valles-Coral et al. (2024): (1) data collection, (2) preprocessing, (3) processing, and (4) model validation.

For the data collection phase, the dataset was obtained from the Universidad Nacional de San Martín, specifically at the university campus in Tarapoto, Peru. A trained team of health professionals used the Rad-67 device to non-invasively measure hemoglobin (Hb) levels, thus optimizing the clinical data capture process. In parallel, another working group was responsible for capturing images of the participants' nails using a Samsung Galaxy A73 5G smartphone, carefully controlling the lighting and angle conditions. These images are stored in a database to facilitate access and initial processing.

In the preprocessing stage, image cleaning and formatting procedures were performed along with augmentation techniques to improve the diversity of the dataset and reduce the risk of overfitting. This included extracting the region of interest from the nail images and center cropping them, ensuring a structured and uniform dataset.

During the processing phase, CNN models were deployed using the DenseNet169, InceptionV3, and Xception architectures, which were selected for their ability to extract relevant visual features from the nail images. These models were trained with a preprocessed dataset, and the key hyperparameters were tuned to maximize the model performance using advanced optimization techniques.

Finally, in the validation phase, models were continuously evaluated by recording performance metrics such as accuracy (Equation 1), precision (Equation 2), recall (Equation 3), F1-score (Equation 4), and area under the ROC curve (AUC) (Equation 5) on the training and validation sets. To ensure the retention of the best-performing model, the ModelCheckpoint technique was employed, which automatically saved the model weights corresponding to the lowest validation loss during training. This approach prevented overfitting and allowed the selection of the most optimal model for the final evaluation. Furthermore, at the end of training, additional metrics were applied to further assess model performance and confirm its generalization capability.

**Figure 1:**
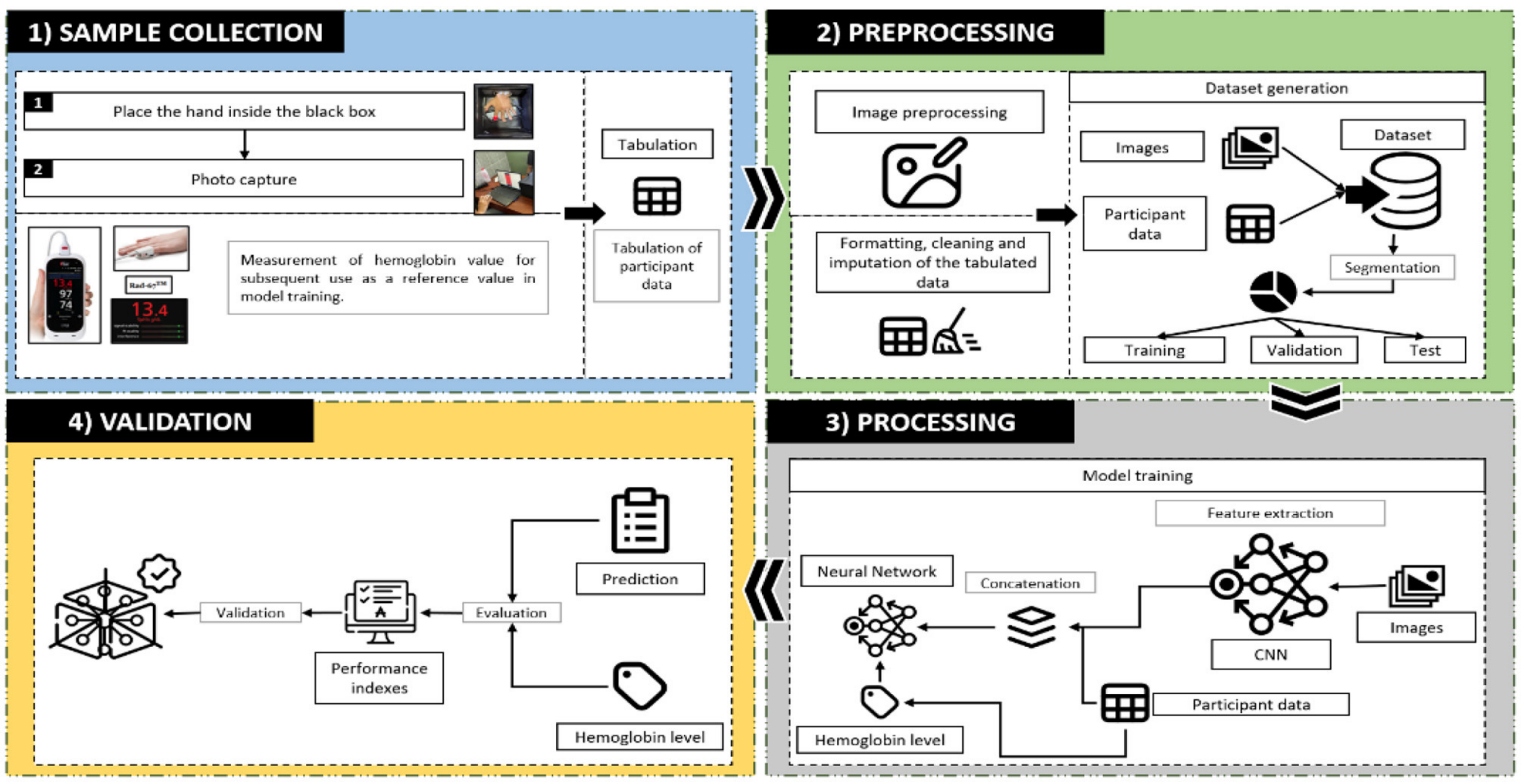
Flowchart of the proposed methodology.

Figure 1 illustrates the methodology of the study, showing the workflow and interconnections between the stages of the research process: data collection, preprocessing, processing, and validation. This block diagram is essential for understanding the sequence and technical approach applied in this research.

### 3.1 Image and clinical data collection protocol

#### 3.1.1 Participants

The study population included 5,575 undergraduate students from the Universidad Nacional de San Martín (UNSM) during the 2023-I academic semester. Non-probabilistic convenience sampling was used to obtain 909 images of students' nails using a standardized collection protocol. The sample size was limited to the number of hemoglobin readings available on the Rad-67 device (up to 1,000 readings). The inclusion criteria included students between 18 and 25 years of age who provided informed consent. The final sample comprised 540 women and 369 men, all of whom met the requirements for participation in the study.

#### 3.1.2 Equipment selection and capture parameter settings

For image capture, the Samsung Galaxy A73 5G was selected for its high 108 MP resolution and manual adjustment capability, which guaranteed optimal quality for subsequent processing and analysis. This device offers stability in capturing data, minimizes distortion, and ensures data consistency. With a storage capacity of 256 GB and a 2.4 GHz Octa-Core processor, it was easy to handle large volumes of images.

The camera was controlled remotely using scrcpy software, which allowed the capture conditions to be standardized in terms of lighting and angle, improving the uniformity of the data collected. To optimize image quality, specific parameters were set: an aperture of *f* /1.8 that allowed adequate light entry and minimized noise, a shutter speed of 1/180 to avoid blurring, a lens size of 5.06 mm to capture the nail surface in detail, and an ISO of 50 that reduced digital noise under controlled lighting conditions inside a polycarbonate box. These settings are essential for accurate analysis using machine vision.

#### 3.1.3 Image capture method

**Figure 2:**
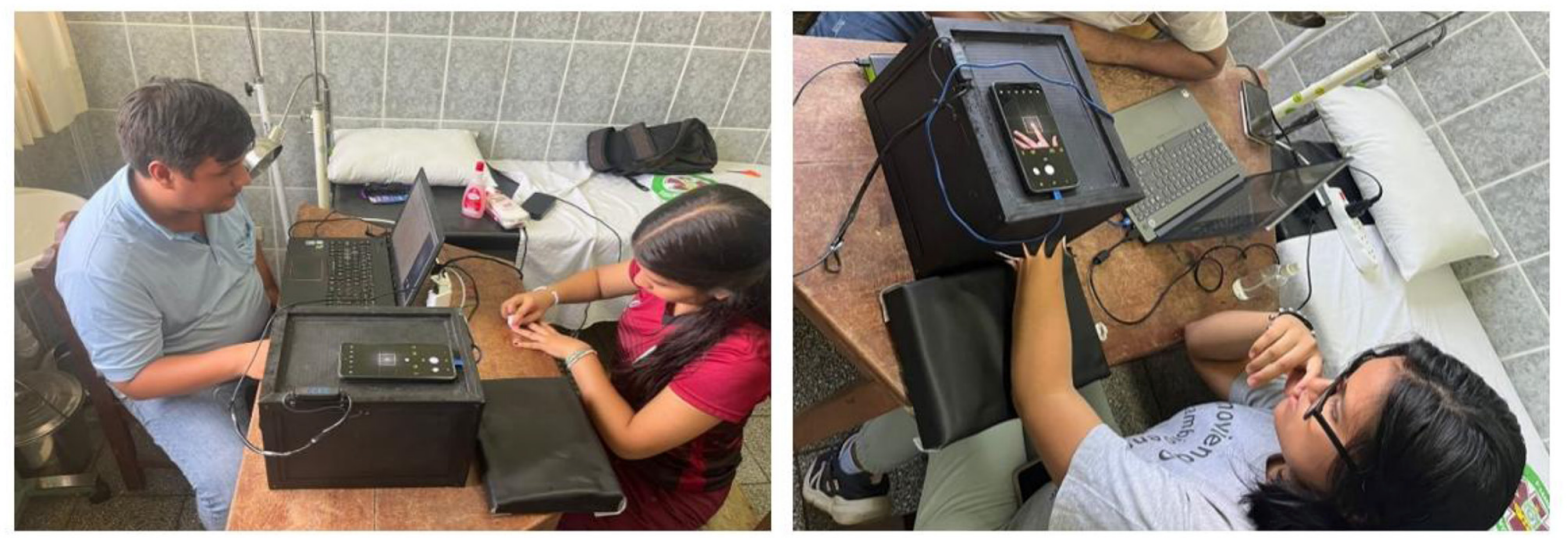
Image capture method.

We developed a detailed protocol for image capture, including specific instructions, to ensure the quality and consistency of the collected data. Each participant was prepared by thoroughly washing and drying their hands before the image capture. The left index finger was then placed in a specially designed polycarbonate box to create a controlled environment, minimizing interference from external light. Finally, a clear image of the index fingernail was captured. During the implementation of the protocol, the participants were instructed to keep their finger still and not to exert pressure on the lens, which was essential to ensure the accuracy of the image in a controlled environment. The procedure is illustrated in Figure 2.

#### 3.1.4 Collection of demographic and clinical data

In addition to these images, we collected detailed demographic and clinical information from each participant using a structured chart. This chart includes identifying data such as name, age, sex, and professional school. Symptoms of anemia experienced in the past month were also recorded, including fatigue, weakness, irregular heartbeat, shortness of breath, dizziness, chest pain, cold hands and feet, and headaches, along with an additional question regarding whether the participant followed a vegetarian or vegan diet. Pulse, respiratory rate, and blood pressure were recorded. Anthropometric data, such as weight, height, body mass index (BMI), and abdominal circumference were also collected. Finally, the results of non-invasive hemoglobin tests performed with the Rad-67 device were included, classifying anemia levels according to standards for women and men, along with heart rate and blood oxygen saturation. These data were organized to facilitate the analysis and correlation with anemia levels detected in the participants.

It is worth mentioning that the data collection process was completed within 2 weeks, focusing on a geolocated sample of university students from a single region. Due to the controlled scope of the study and the structured data collection protocol, no significant temporal or spatial complexity was encountered. The standardized methodology ensured consistency in image capture conditions, clinical measurements, and demographic data collection, minimizing external variability and enhancing the reliability of the dataset.

### 3.2 Preprocessing

In the image preprocessing phase, specific functions were developed to extract and prepare the region of interest, centered on the index fingernail of each participant. The extract_hand function allowed the finger to be isolated from the image background, ensuring that only the relevant region was included in the analysis. This function uses contour segmentation and color thresholding techniques to separate the finger from other visual elements and to ensure accuracy in the selection of the area of interest. Subsequently, a center crop function was designed to center the image and adjust it to a standard size, allowing all images to maintain a uniform and standardized arrangement. This optimized the quality and consistency of the images entered into the computer vision model.

The preprocessed images were converted into numerical arrays for use in deep-learning models. Each image was linked with labels indicating the participant's level of anemia and complemented with additional clinical and demographic data, such as anemia-associated symptoms, weight, height, and sex. This combination allowed the creation of a well-structured dataset in which the images were enriched with relevant clinical information, facilitating the training and validation of the model.

**Figure 3:**
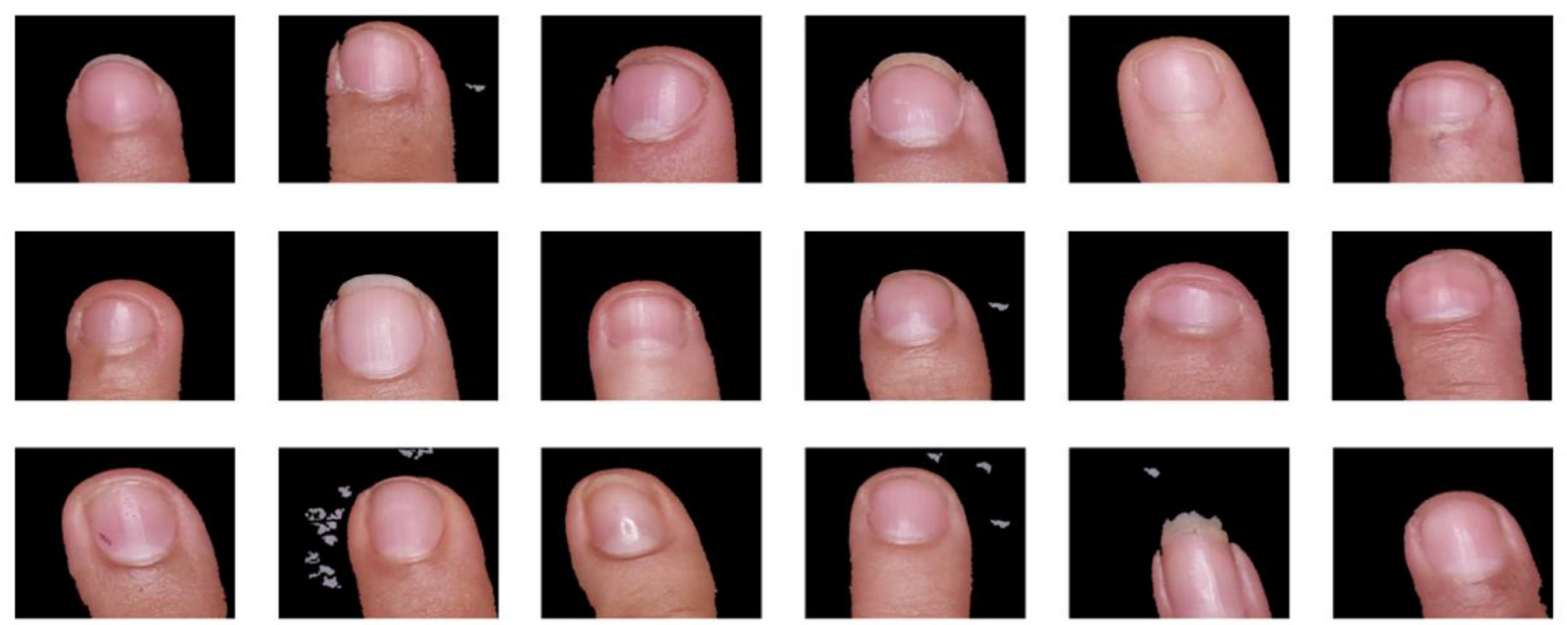
Random sample of preprocessed images.

The results of this preprocessing demonstrated the efficiency of the developed functions. The extract_hand function accurately segmented the finger region, ensuring that only the area of interest was analyzed. The crop_center function centered and resized the images, providing uniformity in the dataset and improving the consistency for the processing of the model. Finally, the structuring of the dataset, in which each image was accompanied by its associated labels and clinical data, resulted in a solid and homogeneous database for the analysis of anemia levels in the studied population. This preprocessing was essential to ensure the quality of the data used in the computer vision model, thus providing a reliable platform for the non-invasive detection of iron deficiency anemia in university students. Figure 3 shows the results of the preprocessing performed.

#### 3.2.1 Consolidation and exploration of the final dataset

In the final stage, all collected data were consolidated into a structured dataset that included the preprocessed images, along with the demographic and clinical information of the participants. This dataset was stored in a format suitable for analysis using computer vision models, allowing the efficient management of the variables in the modeling process.

To understand the general characteristics of the dataset, a descriptive analysis of demographic variables, anemia symptoms, anthropometric data, and physiological measurements was performed. All participants were in the age range of 18–25 years, and the sex distribution included 502 women and 324 men in the 826 profiles valid for training. Participants were classified according to the World Health Organization (2011), differentiating them into anemic and non-anemic groups. Of the profiles evaluated, 238 women and 31 men were identified as having anemia, while 264 women and 293 men did not.

**Figure 4:**
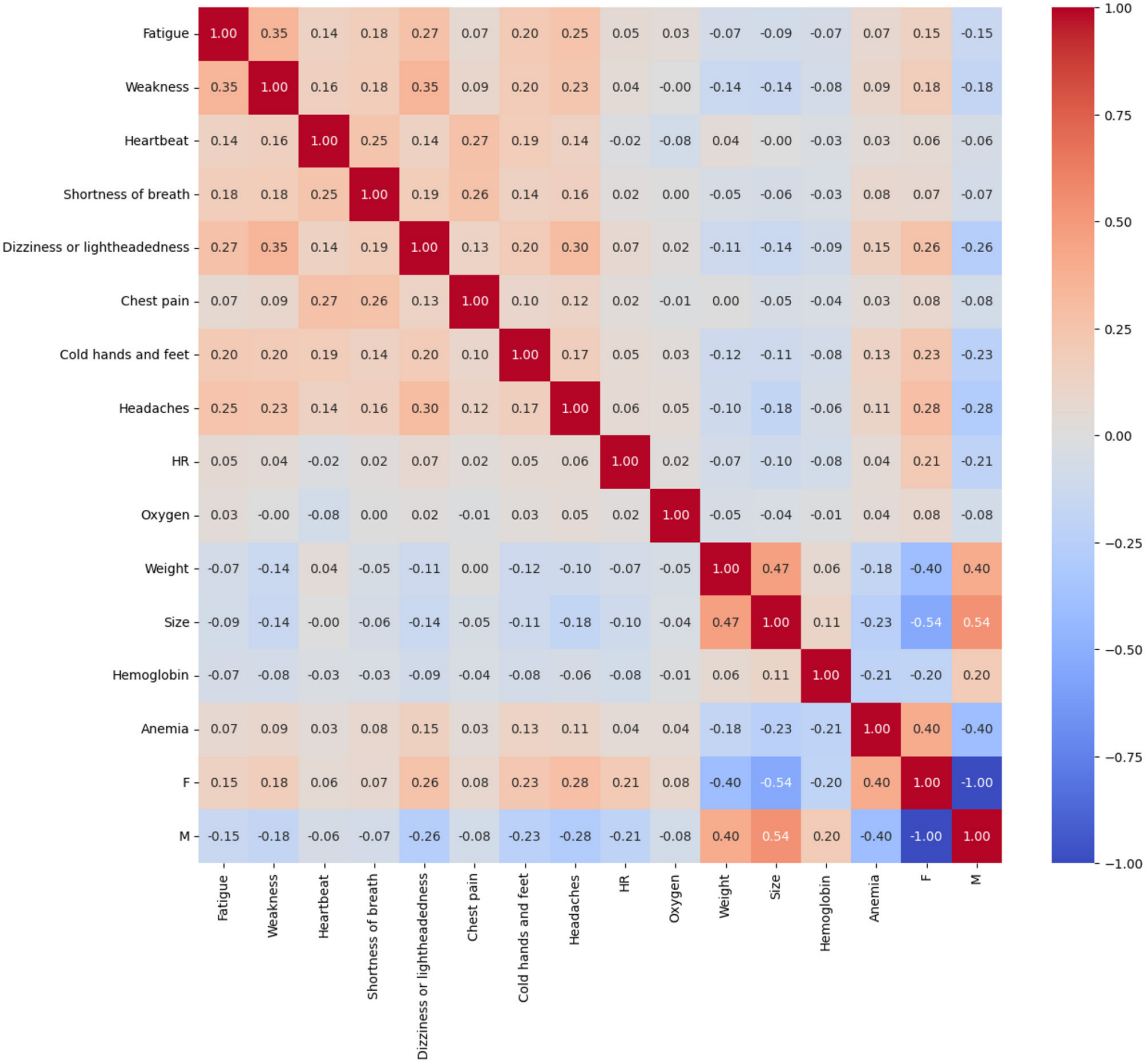
Correlation matrix of symptoms and clinical parameters in the detection of anemia.

Figure 4 presents the correlation matrix that visualizes the relationships between various anemia symptoms and the clinical parameters collected. This matrix, represented by a heat map, uses a color scale that varies from red (indicating strong positive correlations) to blue (indicating strong negative correlations). This visualization provides a clear representation of the interactions and relationships between variables, offering additional information about the clinical characteristics and their possible association with anemia in the population studied.

### 3.3 Processing

To optimize model generalization, we applied advanced preprocessing and data augmentation techniques using Keras ImageDataGenerator. We implemented pixel normalization (rescaling), where each pixel intensity **I** was scaled to the [**0**, **1]** range as **I**′ = **I/255I**. Spatial shifts (width and height shift range = 0.3) were applied using transformations of the form $x^{\prime }=x+t_{X},\;y^{\prime }=y+t_{Y}$, where **t**_**X**_ and **t**_**Y**_ were random translations within 30% of the image dimensions. Random rotations (up to 180°) were performed using the transformation matrix:


$$\begin{array}{l} M=\left[\begin{array}{cc} \cos & -sin\\ \sin & \cos \end{array}\right] \end{array}$$

Where was randomly selected within [−180, 180]. Shearing (shear range = 0.1) was applied using the affine transformation:


$$\begin{array}{l} M=\;\left[\begin{array}{cc} 1 & shear\\ 0 & 1 \end{array}\right] \end{array}$$

with shear values randomly sampled from [−0.1, 0.1]. Random zooming (zoom range = 0.1) was incorporated through the scaling matrix:


$$\begin{array}{l} M=\;\left[\begin{array}{cc} s_{x} & 0\\ 0 & s_{y} \end{array}\right] \end{array}$$

Where *s*_*x*_, *s*_*y*_ were randomly chosen within [0.9, 1.1]. Finally, horizontal flipping was applied by inverting pixel coordinates along the vertical axis as **x**^′^ **=** **W−x**. These transformations expanded the dataset from 826 to 4125 images, enhancing model robustness and generalization.

**Figure 5:**
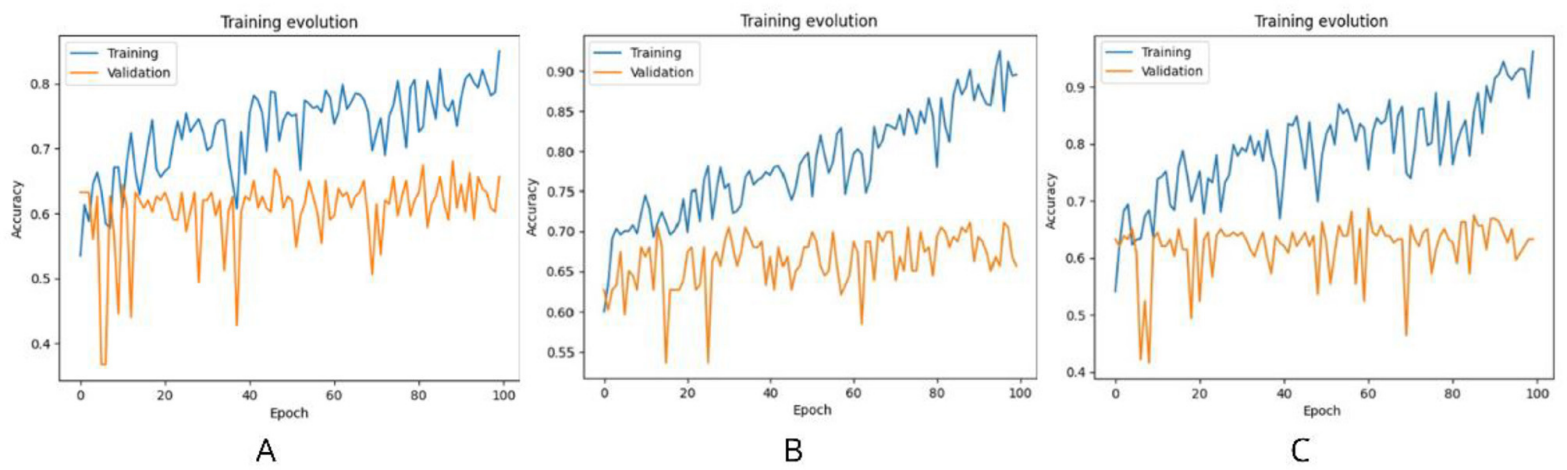
Evolution of training and validation of the models. **(A)** InceptionV3 model showing significant fluctuations in validation accuracy. **(B)** DenseNet169 model with the best overall performance and greater stability. **(C)** Xception model with similar behavior to InceptionV3 but lower stability.

Over 100 training epochs, we monitored the accuracy metrics for both training and validation sets. The InceptionV3 model achieved a maximum validation accuracy of 0.66, although it presented significant fluctuations, indicating the need for additional adjustments to improve its generalization (Figure 5A). In contrast, the DenseNet169 model demonstrated better overall performance, reaching a maximum validation accuracy of 0.69, which positioned it as the most promising model in the context of this study (Figure 5B). Finally, the Xception model showed similar results to InceptionV3, with a maximum validation accuracy of 0.66, although it had lower stability during training (Figure 5C).

After analyzing Figure 5, DenseNet169 stood out as the best-performing model during training, achieving higher accuracies compared to the other architectures evaluated. However, all the models faced significant challenges in terms of generalization, as evidenced by the accuracy values obtained in the validation sets. These limitations suggest that the models could be affected by overfitting issues and insufficient representation of features in the unseen data.

To address these limitations, it is necessary to implement improvement strategies, such as more precise hyperparameter tuning, application of cross-validation to reduce the risk of overfitting, and integration of ensemble models that can combine the strengths of each architecture. In addition, diversifying and balancing the dataset could contribute to a better representation of the variations present in real data, thus increasing the diagnostic accuracy in clinical contexts.

#### 3.3.1 Visual results of processing

**Figure 6:**
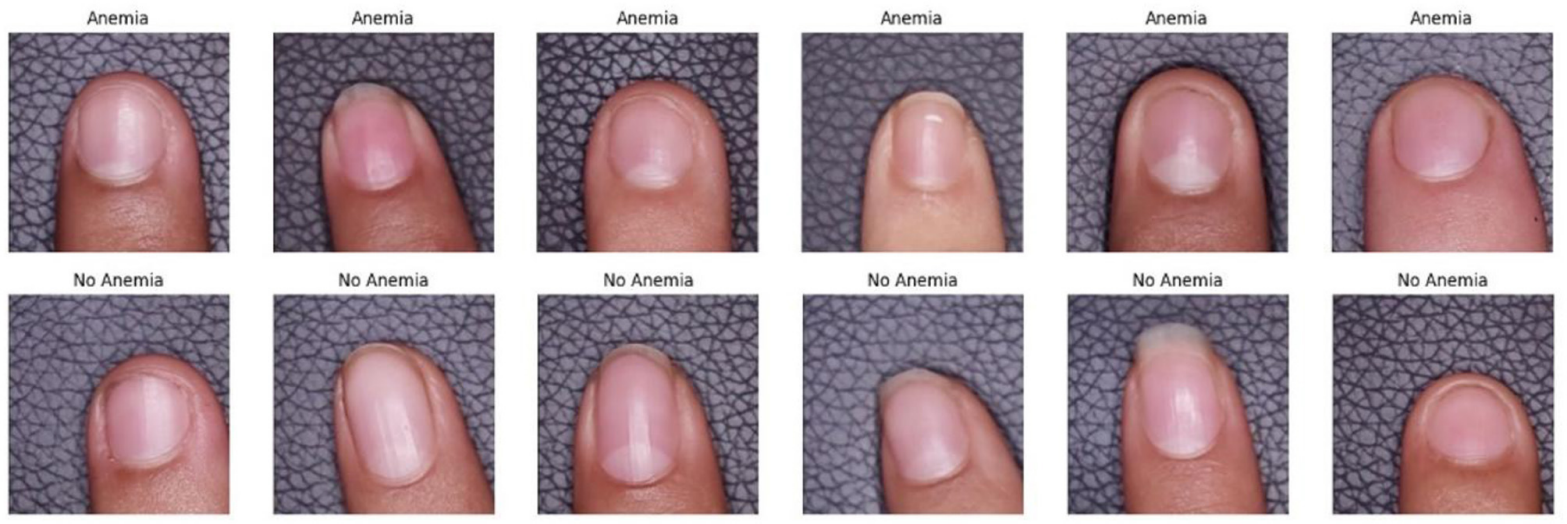
Example of images classified by computer vision models.

A random sample of the results obtained after the image processing is shown in Figure 6. These results illustrate how the models were able to correctly label images of nails classified as having or without anemia, demonstrating the potential of the computer vision tools developed in this study.

The evaluation of the results confirms that the analyzed models are promising, although further adjustments are required to ensure their reliable implementation in clinical settings. These findings represent a significant advance toward the development of non-invasive tools for the early diagnosis of iron deficiency anemia, with potential for their application in health systems based on artificial vision technology.

### 3.4 Validation

Model evaluation is critical in quantifying the performance of a classifier or model. It aims to ensure that the relationships learned from the training dataset are applicable and effective for a validation or test dataset (Rahman Khan et al., 2022). Based on the studies by Valles-Coral et al. (2024) and Appiahene et al. (2023), we selected the following performance metrics:


(1) $$\begin{array}{l} Accuracy\;\left(ACC\right)=\;\frac{TP+TN}{TP+TN+FP+FN} \end{array}$$

(2) $$\begin{array}{l} Precision\;\left(P\right)=\;\frac{TP}{TP+FP} \end{array}$$

(3) $$\begin{array}{l} Recall\;\left(R\right)=\;\frac{TP}{TP+FN} \end{array}$$

(4) $$F1-Score=\frac{2(P*R)}{P+R}$$

(5) $$\begin{array}{l} AUC=\;\frac{TPR-TNR}{2} \end{array}$$

Where TP (True Positives), TN (True Negatives), FP (False Positives), FN (False Negatives), TPR (True Positive Rate), and TNR (True Negative Rate) are standard classification evaluation terms.

## 4 Results and discussion

The performance evaluation of the three deep learning models trained for anemia detection is presented in Table 1. These results provide a direct comparison of accuracy, precision, recall, F1-score, and AUC across the training, validation, and test sets, allowing a structured assessment of each model's classification effectiveness. Among the evaluated architectures, DenseNet169 exhibited the highest overall consistency, followed by Xception and InceptionV3. The following sections provide an in-depth analysis of these findings, focusing on model generalization, potential optimizations, and comparisons with prior research.

**Table 1:** Performance metrics of the evaluated models.

**Model**	**Subset**	**Accuracy (%)**	**P (%)**	**R (%)**	**F1-score (%)**	**AUC (%)**
InceptionV3	Training	72.38	70.12	71.24	70.67	71.43
	Validation	69.15	67.34	68.19	67.72	66.89
	Test	68.07	66.39	67.20	66.58	65.91
DenseNet169	Training	74.23	72.18	68.35	69.27	77.14
	Validation	71.86	70.23	65.84	67.12	75.28
	Test	71.08	69.83	64.77	65.25	74.09
Xception	Training	71.35	69.24	69.18	69.11	71.29
	Validation	69.87	68.12	67.95	68.03	70.14
	Test	68.67	66.30	66.30	66.30	68.36

### 4.1 Performance of the evaluated models

The quantitative analysis of model performance, as shown in Table 1, indicates that DenseNet169 achieved the highest accuracy (74.23% training, 71.86% validation, and 71.08% test) and AUC (77.14% training, 75.28% validation, and 74.09% test), suggesting strong generalization capability and robust classification power. In contrast, InceptionV3 and Xception demonstrated slightly lower performance, with test accuracies of 68.07% and 68.67%, respectively. The AUC values for these models were also lower (65.91% for InceptionV3 and 68.36% for Xception), indicating reduced discriminative ability compared to DenseNet169.

During the training phase, all models achieved high accuracy, with DenseNet169 performing the best (74.23%), followed by InceptionV3 (72.38%) and Xception (71.35%). This suggests that the architectures effectively learned feature representations from the dataset. However, the AUC values indicate that DenseNet169 had a superior ability to distinguish between anemic and non-anemic cases (77.14% AUC), confirming its higher discriminative power compared to the other models.

In the validation phase, a decline in performance was observed, as expected when generalizing to unseen data. DenseNet169 maintained its superiority, achieving 71.86% accuracy and 75.28% AUC, reinforcing its capability to generalize. Conversely, InceptionV3 exhibited the sharpest drop in AUC from 71.43% (training) to 66.89% (validation), indicating potential overfitting. Xception maintained a more stable performance, but its slightly lower recall suggests a reduced sensitivity in identifying anemic cases.

In the test phase, DenseNet169 continued to be the most reliable model, attaining the highest recall (64.77%) and AUC (74.09%), confirming its robust classification performance across datasets. InceptionV3 and Xception, while still competitive, showed greater fluctuations in their ability to correctly classify anemia cases, emphasizing the need for further optimization in feature extraction and generalization strategies.

**Figure 7:**
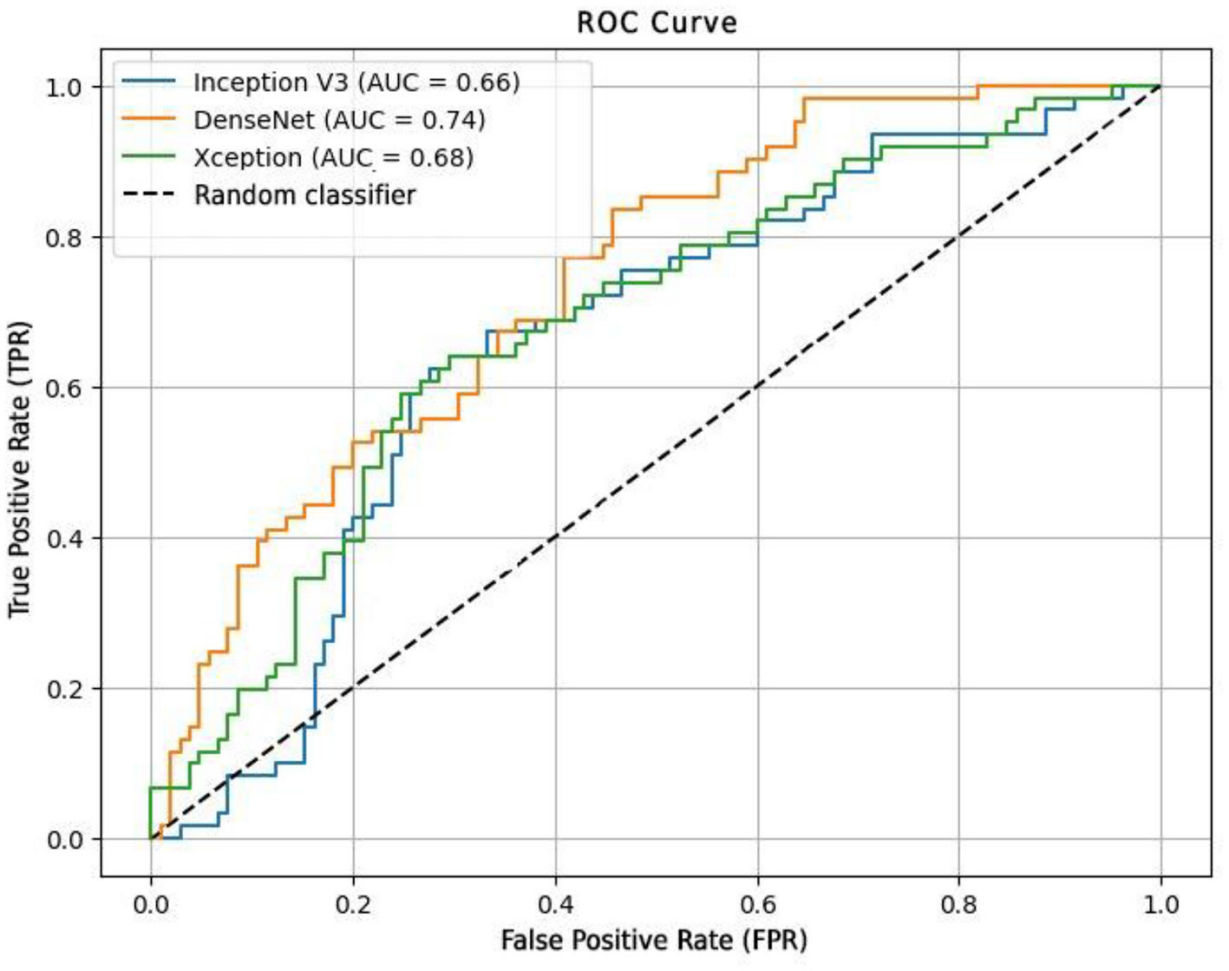
ROC curve for the evaluation of computer vision models.

A more detailed evaluation of model performance in the test phase is presented in Table 2. The results confirm that DenseNet169 maintained the best overall performance, achieving 71.08% accuracy, 69.83% precision, and 74.09% AUC. These metrics indicate that this model was able to effectively distinguish anemic from non-anemic cases while maintaining stable generalization across datasets. In contrast, InceptionV3 showed the weakest classification capability, with 68.07% accuracy and 65.91% AUC, reinforcing its tendency toward overfitting, as observed in the validation phase. Xception performed slightly better, achieving 68.67% accuracy and 68.36% AUC, demonstrating a more balanced trade-off between sensitivity and specificity compared to InceptionV3. This performance is further illustrated in Figure 7, which presents the ROC curves for the evaluated models, highlighting their ability to distinguish between anemic and non-anemic cases.

**Table 2:** Test phase performance of evaluated models.

**Model**	**Accuracy**	**Precision**	**Recall**	**F1-score**	**AUC ROC**
InceptionV3	0.6807	0.6639	0.6720	0.6658	0.6591
DenseNet169	0.7108	0.6983	0.6477	0.6525	0.7409
Xception	0.6867	0.6630	0.6630	0.6630	0.6836

### 4.2 Confusion matrix analysis

**Figure 8:**
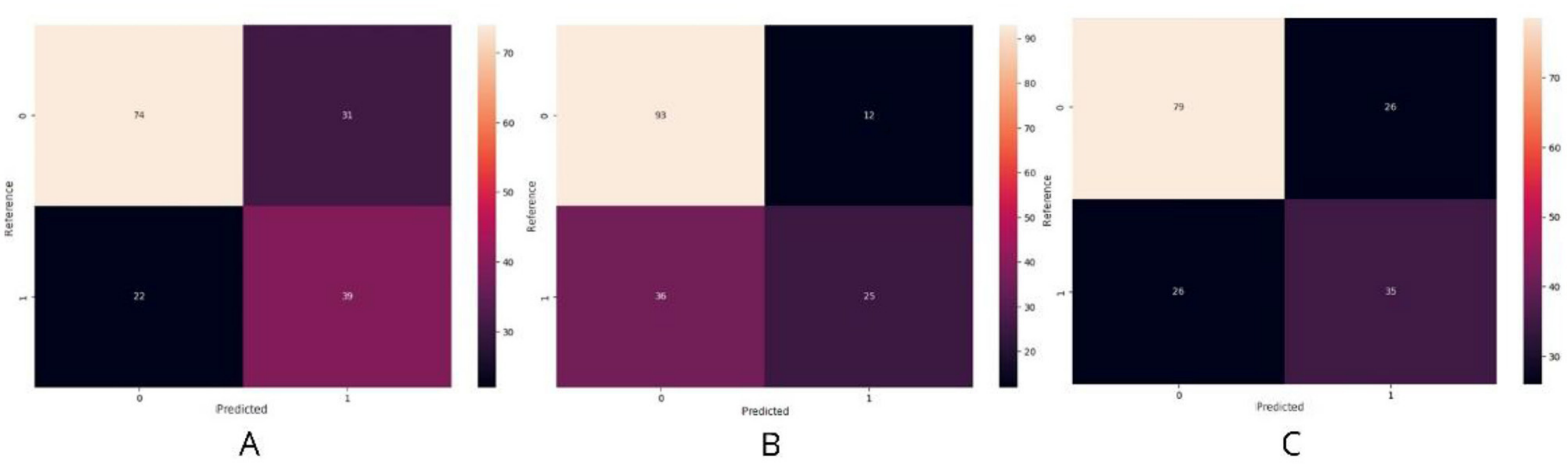
Confusion matrices of the models. **(A)** InceptionV3 model showing an acceptable recall but requiring optimization to reduce misclassifications. **(B)** DenseNet169 achieving the best balance between precision and recall, although false negatives remain a challenge. **(C)** Xception model displaying intermediate results, with opportunities for improvement through parameter tuning and data augmentation.

To further evaluate model effectiveness, confusion matrices were analyzed (Figure 8), providing insights into classification errors and areas for improvement. For InceptionV3, in the validation set, 74 correct classifications were obtained for the negative class compared to 31 incorrect ones, and 39 hits were recorded for the positive class with 22 errors (Figure 8A). This indicates an acceptable recall but suggests a need to optimize parameters to reduce false positives and false negatives.

For DenseNet169, the results were more promising, with 93 correct predictions for the negative class compared to only 12 incorrect ones, while for the positive class, 25 hits and 36 errors were recorded (Figure 8B). These values demonstrate a better balance between precision and recall, though minimizing false negatives remains a key area for improvement.

Xception presented intermediate results, correctly classifying 79 cases in the negative class vs. 26 incorrect ones, and 35 correct answers in the positive class vs. 26 errors (Figure 8C). This suggests a trade-off between recall and precision, where further adjustments, such as hyperparameter tuning or increased data augmentation, could enhance overall performance.

### 4.3 Comparative analysis of non-invasive anemia detection methods

To evaluate the effectiveness of the proposed approach, a comparative performance analysis was conducted against existing non-invasive anemia detection methods (Table 3). The results indicate that while our DenseNet169 model achieved a competitive AUC of 74.09%, it still lags behind some state-of-the-art methods that integrate multimodal inputs or additional hardware components.

**Table 3:** Comparisons of existing methods.

**Method**	**Dataset**	**Metrics**					**Contribution**
		**ACC (%)**	**P (%)**	**R (%)**	**F1 (%)**	**AUC (%)**	
Naive Bayes on nail images (Peksi et al., 2021).	Not specified	87.5–92.3	–	–	–	–	Naive Bayes classified anemia with controlled lighting.
Deep learning with nail data (Yilmaz et al., 2022).	353 participants	–	–	–	–	–	Reliable hemoglobin estimation using deep learning.
IoMT for anemia detection (Ghosal et al., 2022).	99 participants	–	–	90	–	–	Hybrid smartphone model for anemia detection.
CNN for deficiency detection (Selvi et al., 2022).	Images collected from web crawlers	94	–	–	–	–	AI predicted deficiencies from fingernail images.
AI nail pallor analysis (Das et al., 2023).	220 participants	91	–	96	–	–	MLP-based model for hemoglobin estimation.
ML models on fingernails (Asare et al., 2023).	710 images (collected from 10 hospitals)	98.33	97.64	97.44	97.54	99.93	CNN outperformed ML models for anemia.
DenseNet169 on nail images (This Study).	909 images	71.08	69.83	64.77	65.25	74.09	Deep learning improved non-invasive anemia detection.

Das et al. (2023) developed a hybrid approach that combines a smartphone-based imaging system with a custom pressure-sensitive device to induce color changes in the nail bed. Their method achieved an RMSE of 0.63 g/dL by leveraging hemoglobin's dynamic response to mechanical stimulation, enhancing feature extraction. In contrast, our model relies solely on static images, limiting the granularity of extracted features. Future improvements could explore color change modeling through sequential image capture or hyperspectral imaging techniques.

Asare et al. (2023) compared different machine learning models for anemia detection, where CNNs outperformed traditional classifiers, achieving 99.12% accuracy. Their dataset included images of the eye conjunctiva, palm, and fingernails, providing a richer feature space. Our model, which uses only fingernail images, may have reduced generalization. Adding metadata such as age, sex, and clinical history, or analyzing multiple anatomical regions, could enhance classification performance.

Peksi et al. (2021) applied the Naïve Bayes method to detect anemia, achieving an accuracy of 87.5% and improving to 92.3% under optimal lighting. Our DenseNet169 model, with an accuracy of 69.83%, is below these values. This suggests that better image preprocessing, such as YCbCr color space conversion and color feature segmentation, could improve performance.

Yilmaz et al. (2022) used a deep learning approach that combined numerical data with nail images, obtaining low error values and high accuracy in estimating hemoglobin levels. In contrast, our study focused solely on nail images, which may explain differences in accuracy. Combining different data types and specialized models for each could improve predictions. Additionally, their model's fast response time of 0.09 s highlights the potential of these technologies for real-time applications.

Ghosal et al. (2022) implemented an IoMT-based approach using color spectroscopy, achieving an accuracy of ±0.33 g/dL in predicting hemoglobin levels and a sensitivity of 90%. Their use of a smartphone to capture images from the conjunctiva and nails emphasizes the importance of selecting the right anatomical regions.

Selvi et al. (2022) demonstrated that deep learning models, including ResNet and DenseNet, can achieve 94% accuracy in predicting micronutrient deficiencies. More complex models and training with real-time data could significantly improve accuracy. Integrating advanced imaging techniques and metadata analysis could be a key direction for enhancing our model.

Unlike existing models that require multimodal inputs or specialized hardware, our approach prioritizes cost-effectiveness, accessibility, and real-world applicability. This study focuses exclusively on fingernail images for anemia detection, systematically evaluating and comparing state-of-the-art deep learning architectures, including DenseNet169, InceptionV3, and Xception. This targeted approach demonstrates the feasibility of nail-based diagnostics without additional devices. A standardized image acquisition protocol ensures controlled conditions during data collection using a smartphone camera, minimizing variability due to lighting and angles—key challenges in biomedical imaging were environmental factors impact model performance.

A key differentiator of this study is its dataset, one of the first large-scale collections of fingernail images for anemia detection in young adults and university students. Previous research has primarily targeted children, pregnant women, or hospital patients, whereas this study investigates anemia in young adults and its impact on cognitive function and academic performance. The proposed model is optimized for mobile and cloud-based deployment, reducing computational costs and enabling real-time applications in telemedicine and digital health. Fine-tuning transfer learning models enhances adaptability, improving scalability across diverse populations.

DenseNet169 demonstrated the highest discriminative capacity among the tested models, with superior accuracy and AUC. Although effective for non-invasive anemia detection, performance improvements can be achieved by incorporating advanced preprocessing, integrating multimodal data, and optimizing image capture conditions. These enhancements, supported by prior research, form a foundation for refining non-invasive diagnostic technologies.

This study confirms that computer vision models can accurately detect iron deficiency anemia through nail image analysis. The proposed approach validates results obtained with the Rad-67 hemoglobin meter, offering a complementary and non-invasive diagnostic tool for clinical and educational settings. Continuous optimization and validation will further enhance model accuracy, reinforcing its potential for real-world medical applications.

## 5 Limitations of the study

Although the results validate the feasibility of fingernail image-based anemia detection, certain technical constraints must be addressed. Dataset imbalance, particularly in the distribution of anemia severity levels, may have influenced the model's recall performance. Implementing synthetic data augmentation strategies, such as GAN-based image synthesis or domain adaptation techniques, could mitigate this issue.

Another critical aspect is the standardization of image acquisition conditions. Unlike models trained on controlled hospital environments, our dataset was collected under variable lighting conditions, which may introduce noise in feature extraction. Adaptive illumination correction algorithms and photometric standardization techniques could improve model consistency across different acquisition settings.

Regarding computational efficiency, Google Colab TPU acceleration was utilized for training, but real-time inference feasibility remains an open question. Optimizing model compression via quantization-aware training or knowledge distillation could significantly reduce memory footprint and inference latency, making the model more suitable for mobile and embedded healthcare applications.

Lastly, future work should explore hybrid approaches, integrating clinical metadata with deep learning models to enhance diagnostic precision. Combining image-based predictions with non-invasive physiological markers, such as heart rate variability or peripheral oxygen saturation, could improve classification confidence and clinical applicability.

## 6 Conclusions

This study represents a significant advancement in non-invasive iron deficiency anemia detection, leveraging smartphone-captured fingernail images and deep learning techniques. The DenseNet169 model demonstrated reliable classification performance, achieving competitive precision and recall levels, confirming its potential as an accessible and practical alternative to traditional invasive diagnostic methods. The simplicity and adaptability of this approach position it as a promising solution for early anemia detection in various settings, including clinical, academic, and remote healthcare environments.

Furthermore, the study highlights the feasibility of mobile-based artificial vision algorithms, emphasizing their scalability and real-world applicability. Unlike existing approaches that focus on broader clinical populations, this research specifically targets university students, an underrepresented yet vulnerable group in anemia studies. This focus allows for tailored applications in student health monitoring and academic performance impact assessments.

Future work should focus on enhancing model generalization and robustness, exploring customized training for diverse populations and clinical conditions. Additionally, integrating this approach into digital health platforms could enable continuous, real-time monitoring, further extending its impact beyond early diagnosis to preventive healthcare management. Strengthening these aspects will contribute to the widespread adoption of non-invasive anemia detection methods, reducing diagnostic barriers and promoting broader community wellbeing.

## Data Availability

The raw data supporting the conclusions of this article will be made available by the authors, without undue reservation.
